# Converting from face-to-face to postal follow-up and its effects on participant retention, response rates and errors: lessons from the EQUAL study in the UK

**DOI:** 10.1186/s12874-021-01453-0

**Published:** 2022-02-11

**Authors:** Emer Gates, Barnaby Hole, Samantha Hayward, Nicholas C. Chesnaye, Yvette Meuleman, Friedo W. Dekker, Marie Evans, Olof Heimburger, Claudia Torino, Gaetana Porto, Maciej Szymczak, Christiane Drechsler, Christoph Wanner, Kitty J. Jager, Paul Roderick, Fergus Caskey, for the EQUAL investigators

**Affiliations:** 1 Centre for Population Health Sciences, Bristol Medical School, University of Bristol, Bristol, UK; 2 Southmead Hospital, North Bristol NHS Trust, Bristol, UK; 3 UK Renal Registry, Southmead Hospital, Bristol, UK; 4 ERA-EDTA Registry, Department of Medical Informatics, Academic Medical Center, University of Amsterdam, Amsterdam Public Health Research Institute, Amsterdam, The Netherlands; 5 Department of Clinical Epidemiology, Leiden University Medical Center, Leiden, The Netherlands; 6 Renal Unit, Department of Clinical Intervention and technology (CLINTEC), Karolinska Institutet and Karolinska University Hospital, Stockholm, Sweden; 7 Institute of Clinical Physiology, National Research Council, Reggio Calabria, Italy; 8 GOM Bianchi Melacrino Morelli, Reggio Calabria, Italy; 9 Department of Nephrology and Transplantation Medicine, Wroclaw Medical University, Wroclaw, Poland; 10 Division of Nephrology, University Hospital of Wurzburg, Wurzburg, Germany; 11 School of Primary Care Population Sciences and Medical Education, Faculty of Medicine, University of Southampton, Southampton, UK

**Keywords:** Chronic kidney disease, Prospective cohort study, Retention, Follow-up, Response rates, Errors

## Abstract

**Background:**

Prospective cohort studies are challenging to deliver, with one of the main difficulties lying in retention of participants. The need to socially distance during the COVID-19 pandemic has added to this challenge. The pre-COVID-19 adaptation of the European Quality (EQUAL) study in the UK to a remote form of follow-up for efficiency provides lessons for those who are considering changing their study design.

**Methods:**

The EQUAL study is an international prospective cohort study of patients ≥65 years of age with advanced chronic kidney disease. Initially, patients were invited to complete a questionnaire (SF-36, Dialysis Symptom Index and Renal Treatment Satisfaction Questionnaire) at research clinics every 3–6 months, known as “traditional follow-up” (TFU). In 2018, all living patients were invited to switch to “efficient follow-up” (EFU), which used an abbreviated questionnaire consisting of SF-12 and Dialysis Symptom Index. These were administered centrally by post. Response rates were calculated using returned questionnaires as a proportion of surviving invitees, and error rates presented as the average percentage of unanswered questions or unclear answers, of total questions in returned questionnaires. Response and error rates were calculated 6-monthly in TFU to allow comparisons with EFU.

**Results:**

Of the 504 patients initially recruited, 236 were still alive at the time of conversion to EFU; 111 of these (47%) consented to the change in follow-up. In those who consented, median TFU was 34 months, ranging from 0 to 42 months. Their response rates fell steadily from 88% (98/111) at month 0 of TFU, to 20% (3/15) at month 42. The response rate for the first EFU questionnaire was 60% (59/99) of those alive from TFU. With this improvement in response rates, the first EFU also lowered errors to baseline levels seen in early follow-up, after having almost trebled throughout traditional follow-up.

**Conclusions:**

Overall, this study demonstrates that administration of shorter follow-up questionnaires by post rather than in person does not negatively impact patient response or error rates. These results may be reassuring for researchers who are trying to limit face-to-face contact with patients during the COVID-19 pandemic.

**Supplementary Information:**

The online version contains supplementary material available at 10.1186/s12874-021-01453-0.

## Background

Retention of participants is acknowledged as a major challenge in longitudinal follow-up studies, and attrition is more likely amongst older participants and those in poorer health [1]. Observational studies and randomised controlled trials (RCTs) suffered further disruption to recruitment, retention and follow-up during the COVID-19 pandemic, with delays to study reporting [2]*.* Meanwhile, studies employing follow-up approaches compatible with social-distancing guidelines were able to continue data collection. Here, we reflect on transition from a conventional face-to-face approach to one employing postal follow-up, within the UK arm of the EQUAL study (European QUALity Study). This change was made to improve study efficiency through reduced costs and use of resources, while also reducing patient burden. Not only was this approach found to improve falling response rates, but in retrospect it also provided a valuable lens into maintaining follow-up during the pandemic.

EQUAL is a prospective observational study which has recruited people aged over 65 with advanced chronic kidney disease (CKD; with eGFR ≤20 mL/min/1.73m^2^) from 2012 to present [3]. The study initially involved research-staff administering follow-up until 2017 in the UK, but a number of issues including slower than anticipated recruitment and progression to end-stage in the main study necessitated an extension and in 2018, all surviving UK participants were invited to switch from traditional follow-up (TFU) to efficient follow-up (EFU). This employed an abbreviated questionnaire, administered by post with linkage to the UK Renal Registry, which captures survival and quality assurance data for all individuals with CKD and receiving renal replacement therapy in the UK. The analyses presented here aimed to describe the impact of a switch to remote patient questionnaire follow-up on response rates and error rates in the EQUAL Study.

## Methods

### Study design and study population

The EQUAL study developed a prospective cohort of older people with advanced CKD in Germany, Italy, Poland, Sweden, the Netherlands and the UK. The main research question addresses when dialysis should ideally be initiated in elderly participants with CKD, based on uraemic signs and symptoms, and quality of life [4, 5]. Approval was obtained from the Medical Ethical Committees of the national co-ordinating centres and institutional review boards of the participating centres. Written informed consent was obtained from all participants.

Participants ≥65 years of age were included if their eGFR, as estimated by the Modification of Diet in Renal Disease equation, [6] had dropped for the first time to ≤20 mL/min/1.73m^2^ during the previous 6 months. Participants were excluded if the drop in eGFR represented an acute kidney injury, or if they had previously received any form of renal replacement therapy. Recruitment began in 2012, with more than 1700 participants recruited by November 2020. Data on all 506 UK recruits are presented here.

### Data collection

Clinical and demographic data were obtained from medical records and entered in a web-based clinical record form. Baseline data included ethnicity, primary renal disease, co-morbidities and eGFR. A weighted comorbidity score was calculated using the Charlson Comorbidity Index [7]. Physical examination for body mass index (BMI) and subjective global assessment was performed [8].

Until 2018, follow-up was conducted 3–6 monthly (depending on the level of kidney function), wherein staff assisted participants in completing questionnaires collecting symptoms of kidney disease (Dialysis Symptom Index [DSI]) [9], and quality of life (Short-Form 36 [SF-36]) [10]*.* DSI is available in the public domain; SF-36 was developed by the Research and Development Corporation (RAND) for the Medical Outcomes Study, and is available in the public domain [10]. During this “traditional” follow-up (TFU), other questionnaires were administered to participants including the Renal Treatment Satisfaction Questionnaire (RTSQ) [11], Illness Perception Questionnaire (IPQ) [12] and a questionnaire on Decision-Making in kidney disease (DM) which was developed by the EQUAL investigators. A licence was provided from Health Psychology Research Limited for RTSQ, and email permission was provided from the author of IPQ. The Decision-Making in kidney disease questionnaire is included as a supplementary file (Additional File 1).

In September 2018, the EQUAL protocol was amended in the UK to allow linkage to routine healthcare databases and administration of postal questionnaires (EFU). Local research teams contacted surviving participants to seek their consent to take part, with ongoing follow-up administered centrally through the UK Renal Registry. The first round of EFU questionnaires was administered in October 2019. The original questionnaire was amended to contain the SF-12 instead of SF-36, with 12 of the original 36 questions covering all validated indicators of quality of life: physical functioning, physical role, emotional role, bodily pain, social functioning, mental health, vitality, and general health [13]. A licence from QualityMetric Incorporated, LLC was provided for use of the SF-12 questionnaire. In order to shorten the EFU questionnaire, the RTSQ, IPQ and DM questionnaires were not included. Participants were asked to record the date on which they completed the questionnaire, and whether they had assistance from family or healthcare staff. The original questionnaire in TFU included 102 questions and 11 pages; the abbreviated EFU questionnaire included 80 questions and 8 pages. A freepost envelope was included for return of the questionnaire to the UK Renal Registry.

### Data analysis

Six-monthly traditional (for all 504 initial participants) and subsequent efficient follow-up were analysed and compared. Due to the reconsenting process, EFU was viewed as a new study group and hence non-consenters were not included in the outcome statistics. To facilitate comparison, only the DSI and SF-36 (in TFU) and SF-12 (in EFU) questionnaires are presented. Throughout follow-up, response rates are calculated using returned questionnaires as a proportion of surviving invitees – deceased participants were censored. Baseline clinical and demographic data are reported as mean values with standard deviations for normally distributed continuous variables, median values with interquartile ranges (IQRs) for not normally distributed data, and as proportions for categorical variables.

**Fig. 1:**
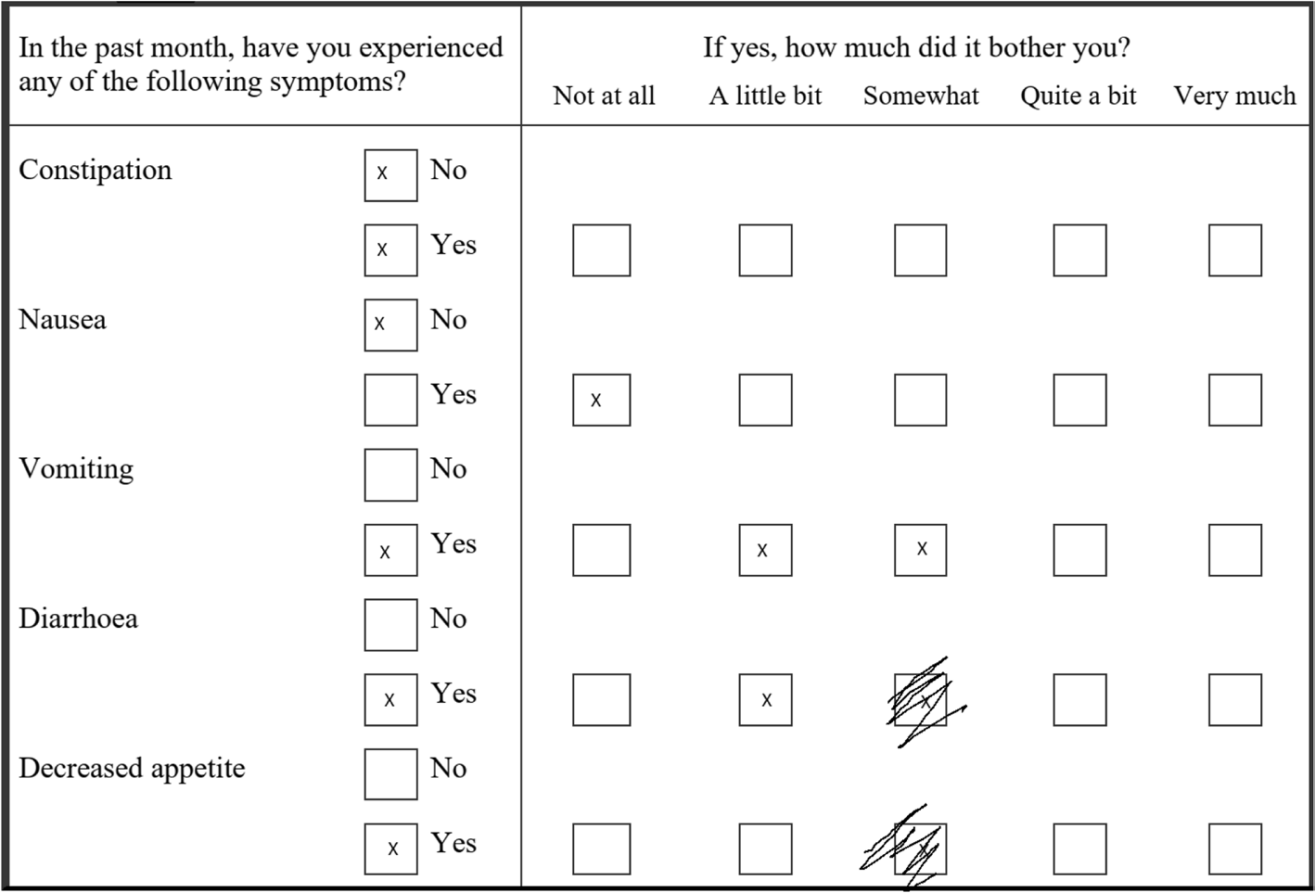
Examples of errors counted in the Dialysis Symptom Index. In the first three questions duplications are shown, with (1) answering both “no” and “yes” simultaneously, (2) answering “no” along with a quantifier for how much the symptom bothers them, and (3) answering two quantifiers together, respectively. The latter two questions show errors related to crossing answers out, with the first example showing a corrected error, and the last example showing an error crossed out but no correction

Errors in EFU responses were coded under the following categories: a missing answer; missed double-page spread of questions; duplication of answers; and crossing answers out. Duplication of answers in the DSI was further clarified into (1) answering both “no” and “yes” for whether they experience the symptom (2); answering “no” alongside how much the symptom bothers them (3); answering ≥2 quantifiers for how much the symptom bothers them. Crossing answers out was further delineated into whether the participant has corrected the cross-out with another answer; or whether they left the crossed-out response uncorrected. Examples of these errors are given in Fig. 1. Although error rates were counted for both follow-up periods, this categorisation was not conducted for questionnaires completed during traditional follow-up. Error rates are presented as the average percentage of unanswered questions or unclear answers, of questions per returned questionnaire (unreturned questionnaires were censored for this calculation).

A comparison was also made for six-monthly TFU between all 504 initial participants and the 236 participants who were alive and invited to participate in EFU, regarding both response and error rates.

## Results

### Characteristics of the study cohort

Of the 506 UK EQUAL participants, 312 (62%) were male and the median age was 76.7 (inter-quartile range [IQR] 70.8–81.7) (Table 1). The majority were white (459, 91%), and the median eGFR was 18.7mls/min/1.73 m2 (IQR 16.5–19.9). A minority (34, 7%) had a university degree, with the majority attaining primary school education only (154, 30%), and 137 (27%) attaining a secondary school education. Most were married (205, 41%). The mean Charlson comorbidity index was 7.0 (standard deviation [SD] 1.8). Just 30 participants had no major co-morbidity listed and co-morbidity data were missing for 15 participants (3%). The most frequent co-morbidities were hypertension, diabetes and malignancy (76, 38 and 21% of individuals respectively). After CKD of unknown aetiology, diabetic nephropathy secondary to type 2 diabetes mellitus was the most common primary renal disease (87, 17.2%).

**Table 1:** Demographic and clinical characteristics of the study cohort

Characteristics	All UK EQUAL participants (***n*** = 506^**a**^)	Alive and invited to EFU (***n*** = 238^a^)	Consented to EFU (***n*** = 113^**a**^)	Did not consent to EFU (***n*** = 125)	Responded to EFU (***n*** = 60^a^)	Did not respond to EFU (***n*** = 41^a^)	Died before EFU survey (*n* = 12)
**Age, median (IQR) years**	76.7 (70.8–81.7)	74.7 (69.0–80.4)	75.1 (68.9–80.5)	74.3 (70.0–79.8)	73.2 (68.2–79.7)	77.0 (70.5–81.1)	76.6 (71.0–82.5)
**Sex,** ***n*** **(%)**							
Male	312 (62)	154 (65)	75 (66)	79 (63)	35 (58)	29 (71)	11 (92)
Female	192 (38)	82 (35)	36 (32)	46 (37)	24 (40)	11 (27)	1 (8)
**Ethnicity,** ***n*** **(%)**							
White	459 (91)	215 (90)	107 (95)	108 (86)	57 (95)	38 (93)	12 (100)
Black: Caribbean	10 (2)	3 (1)	1 (1)	2 (2)	0	1 (2)	0
Black: African	24 (5)	12 (5)	2 (2)	10 (8)	1 (2)	1 (2)	0
Black: (other)	5 (1)	4 (2)	0	4 (3)	0	0	0
Asian: Chinese	6 (1)	2 (1)	1 (1)	1 (1)	1 (2)	0	0
**Marital status,** ***n*** **(%)**							
Married/ living together	205 (41)	116 (49)	64 (57)	52 (42)	34 (57)	23 (56)	7 (58)
Divorced/ separated	29 (6)	17 (7)	9 (8)	8 (6)	7 (12)	0	2 (17)
Widowed/ partner has died	90 (18)	43 (18)	17 (15)	26 (21)	9 (15)	6 (15)	2 (16)
Never married/ lived with partner	21 (4)	9 (4)	6 (5)	3 (2)	2 (3)	3 (7)	1 (8)
Blank	159 (31)	51 (21)	15 (13)	36 (29)	7 (12)	8 (20)	0
**Educational Class,** ***n*** **(%)**							
Primary school	154 (30)	84 (35)	40 (35)	43 (34)	19 (32)	18 (44)	3 (25)
Secondary school or vocational course	137 (27)	78 (33)	46 (41)	32 (26)	26 (43)	12 (29)	8 (67)
University degree	34 (7)	15 (6.5)	8 (7)	7 (6)	6 (10)	2 (5)	0
Other	3 (1)	1 (0.5)	1 (1)	0	0	1 (2)	0
Unanswered	176 (35)	59 (25)	16 (14)	43 (34)	8 (13)	7 (17)	1 (8)
**BMI (kg/m2), mean ± SD**	29.2 (23.6–34.8)	29.4 (23.7–35.1)	29.5 (23.6–35.4)	29.2 (23.8–34.7)	30.2 (23.4–37.0)	29.4 (24.3–34.4)	27.0 (23.2–30.9)
**eGFR, median (IQR)**	18.7 (16.5–19.9)	19.0 (17.0–20.0)	19.0 (17.0–20.0)	19.0 (17.0–20.0)	18.8 (17.0–20.0)	19.0 (16.9–20.0)	18.8 (15.0–20.0)
**PRD,** ***n*** **(%)**							
Glomerular disease	34 (7)	15 (6)	11 (10)	4 (3)	9 (15)	2 (5)	0
Tubulo-interstitial disease	49 (10)	31 (13)	16 (14)	15 (12)	10 (17)	5 (12)	1 (8)
Systemic disease affecting the kidney	21 (4)	10 (4)	5 (4)	5 (4)	2 (3)	2 (5)	1 (8)
Diabetes	99 (20)	44 (18)	19 (17)	25 (20)	8 (13)	10 (24)	1 (8)
Hypertension	68 (13)	35 (15)	15 (13)	20 (16)	9 (15)	4 (10)	2 (17)
Familial/ hereditary nephropathies	12 (2)	6 (3)	2 (2)	4 (3)	1 (2)	1 (2)	0
Miscellaneous renal disorders	104 (21)	45 (19)	21 (19)	24 (19)	8 (13)	9 (22)	4 (33)
Unknown/ missing	117 (23)	50 (21)	22 (19)	28 (22)	12 (20)	7 (17)	3 (25)
**Years since diagnosis, median (IQR)**	3.0 (1.0–6.0)	2 (1–6)	2.0 (1.0–6.0)	3.0 (1.0–7.5)	2.5 (1.0–6.0)	2.0 (1.0–4.3)	2.5 (1.0–4.3)
**Co-morbidities, n (%)**							
Diabetes	193 (38)	81 (34)	31 (27)	50 (40)	14 (23)	14 (34)	3 (25)
Hypertension	386 (76)	179 (75)	81 (72)	98 (78)	40 (67)	30 (73)	11 (92)
History of Major Vascular Event	194 (38)	83 (35)	28 (25)	55 (44)	12 (20)	12 (29)	4 (33)
Malignancy	110 (22)	45 (19)	20 (18)	25 (20)	10 (17)	8 (20)	2 (17)
Blank	15 (3)	5 (2)	4 (4)	1 (1)	3 (5)	1 (2)	0
**Charlson Index, mean ± SD**	7.0 (5.2–8.8)	6.63 (4.91–8.36)	6.4 (4.7–8.0)	6.9 (5.1–8.6)	6.1 (4.4–7.8)	6.6 (5.0–8.2)	6.8 (4.9–8.6)

History of major vascular event includes stroke, myocardial infarction, heart failure and amputation due to peripheral vascular disease. ^a^Initial demographic and clinical details were unavailable for two participants joining in EFU; one who responded and one who consented but did not respond to EFU

Of the 125 people who did not consent to EFU, a higher proportion than in the consenting group were of Black African ethnicity (8% Vs. 2%), and more were widowed (21% Vs. 15%). The majority of participants who did not consent to EFU had a primary renal disease (PRD) diagnosis of diabetes (20%), whereas the majority of those consenting to EFU had an unknown/missing primary renal disease (19%).

The 41 participants who consented to EFU but did not respond were older (77.0 [IQR 70.5–81.1] Vs. 73.2 [IQR 68.2–79.7]), a greater proportion were male (71% Vs. 58%) and had lower levels of post-primary education (36% Vs. 53%) than responders (Table 1).

### Response rates during follow-up

**Fig. 2:**
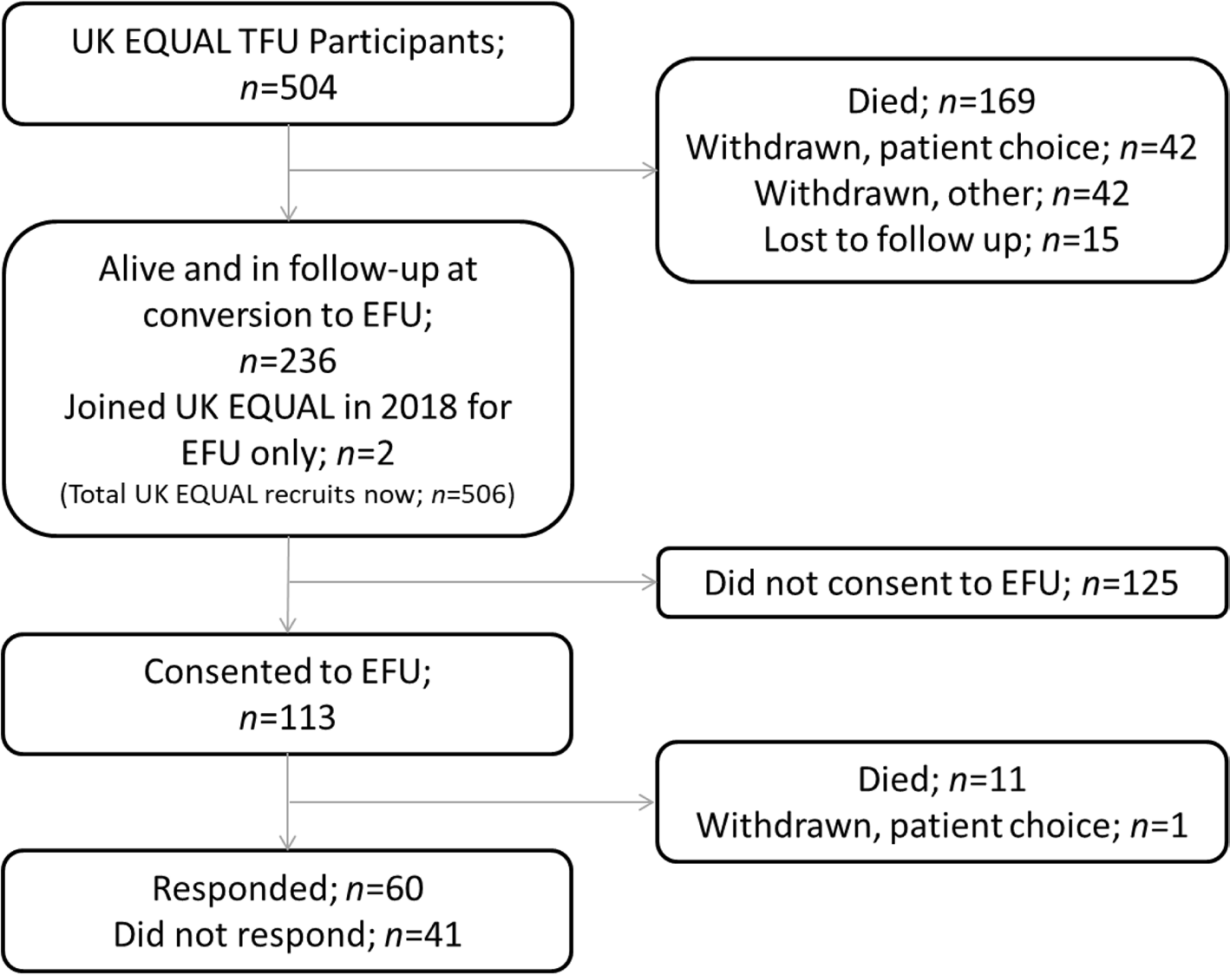
Flowchart showing status of participants throughout EQUAL study.

Of the 504 original UK recruits, 236 were alive and participating in the study on September 1st 2018 and 111 consented to EFU. An additional 2 participants joined the study in 2018 and consented to EFU, totalling 113 participants consenting to EFU out of 238 invited (48%). 125 did not consent to continuing with the EQUAL study in EFU. Eleven consenting individuals died, and one withdrew before the first EFU questionnaire was administered in October 2019. Of the 101 recipients, 60 responded and returned a questionnaire (59%) (Fig. 2).

#### Traditional follow-up – all participants (inclusive of consenters and non-consenters to subsequent efficient follow-up)

As participants were serially recruited between 2013 and 2017, the duration of TFU ranged from 0 to 48 months with a median TFU for the 504 participants of 26.8 months. Four participants died or withdrew after consenting, before returning their first questionnaire, leaving 500 patients to respond at month 0.

**Fig. 3:**
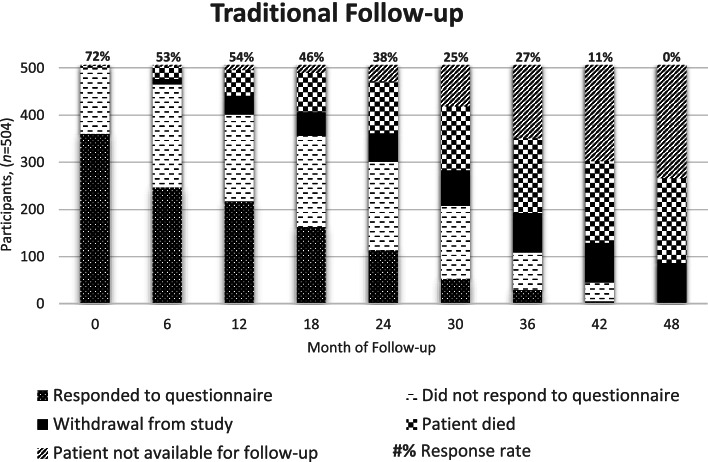
Status of all 504 participants across traditional follow-up. Status includes those who respond or do not respond to each questionnaire, and reasons for withdrawal. “Withdrawal from study” includes patient choice, being discharged from the renal clinic to their GP, moving to a renal centre not involved in the EQUAL study, or receiving a transplant. “Patient not available for follow-up” indicates how long patients have been followed up upon reaching conclusion of traditional follow-up in 2017. Percentages above each bar indicate response rates for participants still in the study at that timepoint

Response rates were calculated at each time point for people who were alive and had not finished follow-up or withdrawn from the study. Response rates throughout TFU for the 504 participants are shown in Fig. 3, with the status of all 504 participants shown at each timepoint, meaning the response rates at each timepoint are directly comparable. Response rates fell gradually every 6 months from 360/500 (72%) at baseline to 52/208 (25%) at 30 months. For those who were recruited earlier and hence took part in follow-up for longer, response rates continued to decline steadily to 0/2 (0%) at 48 months.

#### Follow-up for participants consenting to efficient follow-up (from traditional follow-up through to efficient follow-up)

As described above, in 2018 the remaining EQUAL participants were invited to consent to EFU, with 111 TFU participants opting to continue in the study. EFU was on average 29 months after the participants’ last TFU visit. Of the 111 TFU participants consenting to EFU, median TFU was 34 months, ranging from 0 to 42 months.

**Fig. 4:**
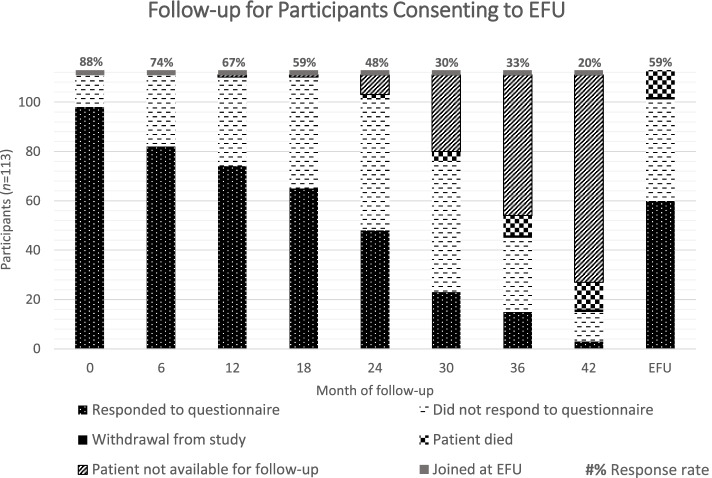
Status of 113 participants throughout TFU and EFU for those who consented to EFU. “Patient not available for follow-up” indicates how long patients have been followed up upon reaching conclusion of TFU in 2017, and subsequently joining EFU in this cohort. Percentages above each bar indicate response rates for participants still in the study at that timepoint

Focussing on TFU response rates for the subset of individuals who consented to EFU, response rates gradually decreased from 98/111 (88%) at baseline, to 23/76 (30%) at 30 months, which was the median end of this cohort’s TFU. Of participants who were recruited earlier to the study and therefore had longer TFU, response rates fell further to 3/15 (20%) at 42 months of traditional follow-up. As two individuals joined the study at the point of converting to EFU in 2018, 113 participants consented to EFU in total. Eleven of these individuals died and one withdrew prior to administration of the questionnaire to the remaining 101 participants. Figure 4 shows the response rates at each timepoint of TFU through to EFU for the 113 participants who consented to EFU. The status of all 113 participants is shown at each timepoint, making the response rates at each timepoint directly comparable. The introduction of the EFU questionnaire appears to boost response rates in this cohort from 30% at median end of TFU (30 months), to 59% (60/101) (Fig. 4).

To investigate whether patients who consented to EFU were better responders throughout or not, comparisons were made for their responses throughout TFU. Response rates throughout TFU were consistently higher in those who responded to the EFU questionnaire, compared with those who did not (Table 2).

**Table 2:** Comparison of response rates throughout follow-up based on response or non-response to EFU

Timepoint, in months	EFU Response	Responded (%)	Difference in response rates (%)
0	Responder	90.0	**7.0**
	Non-responder	83.0	
6	Responder	76.7	**8.7**
	Non-responder	67.9	
12	Responder	70.0	**9.6**
	Non-responder	60.4	
18	Responder	68.3	**23.1**
	Non-responder	45.3	
24	Responder	48.3	**12.5**
	Non-responder	35.8	
30	Responder	21.7	**2.8**
	Non-responder	18.9	
36	Responder	13.3	**0.1**
	Non-responder	13.2	
42	Responder	3.3	**1.4**
	Non-responder	1.9	

### Error rates during follow-up

**Fig. 5:**
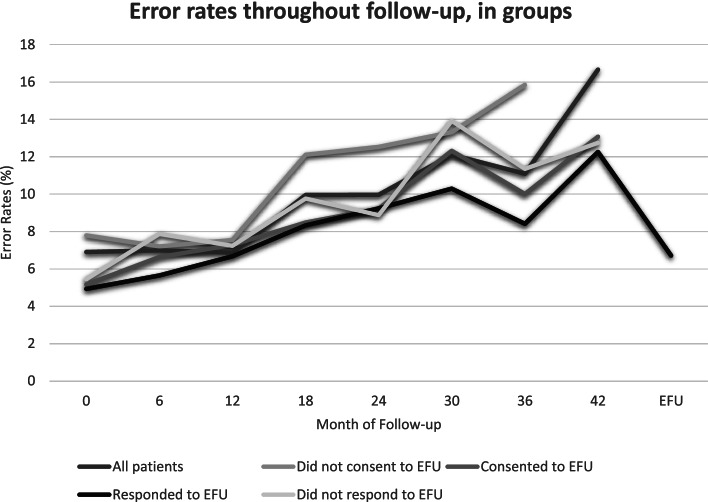
Graph comparing error rates throughout follow-up between groups of EQUAL participants. Error rates are the average percentage of unanswered questions or unclear answers, of questions per returned questionnaire (unreturned questionnaires were censored)

Figure 5 is stratified to show sub-groups of responders and how their error rates reduced after introduction of EFU. In TFU across all 504 participants, error rates increased from 7% at baseline through to 18% at month 42. Error rates in the subset who consented to EFU were comparable to all 504 original participants, rising from 5% at baseline to 13% at month 42 (Fig. 5 and Additional file 2).

Most participants, 51/60 (85%), filled in the EFU questionnaire without assistance, with the remaining nine seeking help from a family member. Nevertheless, at introduction of EFU, errors were comparable with early TFU at 7%, and did not differ substantially between those who had help (5%) compared with those who completed the questionnaire alone (7%). From month 18 onwards, the participants who responded to EFU had the lowest error rates, with highest error rates in those who did not consent to EFU (Fig. 5). Age and duration of follow-up did not appear to have a significant bearing on the number of errors.

### Categories of errors in the EFU questionnaire

The total number of errors made across all 60 questionnaires was 322. The most common error was leaving a single question blank (144/322, 45%). This was followed by errors specific to the DSI questionnaire, where when asked whether they have experienced a particular symptom in the past month, participants answered “no” as well as how much the symptom bothers them, which is only meant to be used for clarifying when answering “yes”, accounting for 23% (74/322) of all errors made. In 20% of errors (65/322), participants failed to complete whole double-page spreads of questions. Other categories of error were less frequent, including four cases where participants wrote or explained that questions regarding sexual function were not applicable (“N/A”) to them (Table 3).

**Table 3:** Categories of errors made in the EFU questionnaire

Category of Error	Number of errors	Percentage of all errors made (%)
Blank	144	44.7
Duplication: “no” along with a quantifier	74	23.0
Skipped whole page	65	20.2
Corrected error	23	7.1
Duplication: Ticked “no” and “yes”	4	1.2
Duplication: ticked 2+ quantifiers	4	1.2
Uncorrected error	4	1.2
Responded “N/A” regarding sexual health	4	1.2
Total	322	100

Labels regarding “Duplication” are related to the Dialysis Symptom Index, whereby some patients ticked “no” for whether they have a particular symptom but also ticked how much the symptom bothers them (“quantifier”), or answered both “no” and “yes” for whether they experience the symptom, or answered ≥ 2 quantifiers for how much the symptom bothers them

No significant difference was seen in TFU response and error rates between the 504 initial participants and the 236 participants who were alive and invited to consent to EFU.

## Discussion

Our work demonstrates that the transition from conventional face-to-face research follow-up to postal follow-up resulted in increased response rates and reduced error rates, in an older cohort of people with advanced CKD. Despite falling response rates and rising error rates, a new focus on the follow-up, even remote, postal follow-up, can achieve response rates and error rates similar to baseline levels. Our findings are timely, as the global COVID-19 pandemic has meant that research studies with traditional clinic-based follow-up have had to pause in the interest of safety, whereas those with remote follow-up have succeeded in continuing with their research [2]. Investigators who are considering altering their study follow-up should be reassured by our results, which illustrate that a change to postal follow-up during an established observational study is feasible and may improve response rates and error rates.

Follow-up fatigue” and attrition are a common problem in research studies, with attrition rates varying from 5 to 70% [14]. Indeed, we observed this attrition during TFU in EQUAL, with participant response rates decreasing from 72 to 25% at 30 months. Maintaining a good participant response rate throughout study follow-up is important but difficult to achieve. There are many potential reasons why people may not complete a research study, including the perceived time burden, feeling under-valued by the researcher, and not being aware of how they are contributing and helping the medical community [15, 16]. The time burden associated with research may be a particularly significant factor for people with advanced CKD, whose clinical care requires frequent interactions with health care professionals for dialysis education, dialysis access investigations and procedures, alongside other speciality appointments to manage their co-morbidities. In addition, older, multi-morbid patients may be dependent on others to help them attend hospital appointments, making face-to-face follow-up less achievable [17].

The improved questionnaire response rates that we observed in EFU may be due to the shorter questionnaire, a preference for remote follow-up to avoid the burden of clinic visits, or due to the novelty of a change in follow-up. In a trial which directly compared different questionnaire administration methods in a cohort of older people, postal follow-up had higher response rates than face-to-face questionnaire administration approaches [18]. Also, research studies with older participants should not discount the possibility of using digital follow-up. In a German study of participant-reported outcomes in breast cancer, when asked about their preferences between digital or paper-based follow-up, a greater proportion of women from all age-groups preferred digital, including 87% of women aged 70–80 [19]. Indeed, studies which collected follow-up data in mobile phone apps found that the older people were the most highly engaged respondents [20, 21]. These findings may be contrary to the perceived stereotypes of older people.

A key problem with attrition in observational studies is that it can introduce bias, as participants who drop out from studies tend to differ from those who continue to participate [22]. People are more likely to withdraw from longitudinal studies if they are older, cognitively impaired, living alone and not married, have a lower socio-economic status or level of education, and/or are less socially active [22, 23]. Furthermore, people with deteriorating health or high symptom burden may be less likely to complete follow-up, thus skewing the final results [24, 25]. We noted that out of those who did not respond to EFU questionnaires, a greater proportion were older, co-morbid males, with lower educational levels. The disparity between males and females has been identified in many research studies, whereby women appear more willing to be involved and continue to respond [20, 26, 27]. The relationship between non-response and educational level is less well established; in contrast to our results, having higher levels of education have been found to correspond to non-response in some longitudinal studies [28]. Although the transition to EFU in our study improved response rates and so should reduce attrition bias, the change required all participants to be re-consented. It is widely recognised that people who consent to participate in research are different to those who do not [29–31]. Indeed, our results show that a greater proportion of people who consented to EFU were white and married when compared to those who did not consent. Therefore, with only 47% of people consenting to EFU, this additional re-consent step should be acknowledged as another stage in which bias could be introduced into the study and affect the external validity of the results.

“Follow-up fatigue” was also demonstrated by a consistent increase in questionnaire error rate in all participants during TFU, regardless of whether they consented or responded to EFU. We observed that introducing EFU halved the error rate and that the majority of participants completed the EFU questionnaire without assistance. This was an unexpected finding as we had anticipated that our older multimorbid cohort may have relied on the research nurse to guide them through the questionnaire, however this finding is consistent with other studies [18]. A plausible explanation is that participants feel more relaxed and under less time pressure when they complete the questionnaire at home and therefore make fewer errors. The most common error in the EFU questionnaire was leaving a question blank, and the beginning of the SF-12 questionnaire had the highest error rate. This is worth considering when designing remote questionnaires, to perhaps include more guidance for participants at the start of the questionnaire.

Our work has demonstrated that a pragmatic change in follow-up did not negatively impact questionnaire response or error rate, instead it appears to have had a positive effect. The main limitation of our work is that the questionnaire used in EFU included the SF-12, rather than the SF-36 which was used during TFU. Although the SF-12 is still a validated questionnaire which includes the 12 questions to cover all quality of life indicators, it is possible that the improvement which we observed in response rates may be due to the administration of a shorter questionnaire [32, 33]. Furthermore, the EQUAL study in the UK was converted to postal follow-up for efficiency and to facilitate longer follow-up without the availability of research nurses, rather than to permit direct comparisons between traditional clinic-based and remote forms of follow-up. As such, our work does not include any qualitative data on the participants’ opinion of the change in follow-up. In order to fully evaluate this, bespoke work would be necessary to compare different forms of follow-up, ideally including digital follow-up approaches. Finally, in our work we did not include a cost analysis, which is another key factor to consider when planning the follow-up strategy in a research study.

## Conclusions

In conclusion, in this well-designed longitudinal cohort study of older participants with advanced CKD, response rates fell and error rates rose throughout clinic-based follow-up. With the introduction of a shorter postal questionnaire, response and error rates improved to levels resembling early follow-up in the study, and some participants responded despite not engaging towards the end of traditional follow-up. This suggests that even in older people with advanced CKD, altering the follow-up approach to post is acceptable and may provide more complete data than traditional follow-up. This is acutely relevant in this period of limited contact in the COVID-19 pandemic, as it provides hope that investigators can continue their follow-up studies remotely without sacrificing participant retention or accuracy of their responses.

## Supplementary Information

**Additional file 1: **Decision-making in kidney disease questionnaire.

**Additional file 2: **Infographic showing the content (black) and error rate (red) throughout the EFU patient questionnaire.

## Data Availability

The datasets used and analysed during the current study are available from the corresponding author on reasonable request.

## Abbreviations

BMI: Body mass index
CKD: Chronic kidney disease
COVID-19: SARS-CoV-2 Coronavirus
DM: Decision-making in kidney disease
DSI: Dialysis symptom index
EFU: Efficient follow-up
eGFR: Estimated glomerular filtration rate
EQUAL: European Quality
ID: Identity
IPQ: Illness perception questionnaire
IQR: Inter-quartile range
IRAS: Integrated Research Application System
LLC: Limited Liability Company
PRD: Primary renal disease
RAND: Research and Development Corporation
RCT: Randomised controlled trial
RTSQ: Renal treatment satisfaction questionnaire
SD: Standard deviation
SF-12: Short-form 12
SF-36: Short-form 36
SGA: Subjective global assessment
TFU: Traditional follow-up
UK: United Kingdom
