# Supplementary material for: Converting from face-to-face to postal follow-up and its effects on participant retention, response rates and errors: lessons from the EQUAL study in the UK
Source: BMC Med Res Methodol. 2022 Feb 11;22:44. doi: 10.1186/s12874-021-01453-0 (PMC8832416; doi:10.1186/s12874-021-01453-0)
Supplement: Supplementary file 1 — Additional file 1: Decision-making in kidney disease questionnaire. [file 12874_2021_1453_MOESM1_ESM.pdf]

## Decision-making

## To what extent do you agree with each of the follow statements in relation to your kidney disease?

**1. I feel like I have been given all the information I need about my medication and diet**

☐ Disagree strongly ☐ Disagree ☐ Neither agree nor disagree ☐ Agree ☐ Agree strongly

**2. The possibility of requiring dialysis treatment in the future has been discussed with me**

☐ Yes ☐ Yes, but I did not want* to talk about this ☐ No*

(* Please, proceed to question 8 )

**3. I have been given enough information to be involved in the decision when to start dialysis**

☐ Disagree strongly ☐ Disagree ☐ Neither agree nor disagree ☐ Agree ☐ Agree strongly

**4. I have been fully involved in the decision about the best timing to start dialysis**

☐ Disagree strongly ☐ Disagree ☐ Neither agree nor disagree ☐ Agree ☐ Agree strongly

**5. Could you indicate from the following options how much information you have received from your doctor or nurse? (1=, 5=very much information)**

Hemodialysis:

☐ No information ☐ Little information ☐ Neither a lot nor a little ☐ A lot information ☐ Very much information

Peritoneal dialysis:

☐ No information ☐ Little information ☐ Neither a lot nor a little ☐ A lot information ☐ Very much information

What happens if I choose not to have dialysis:

☐ No information ☐ Little information ☐ Neither a lot nor a little ☐ A lot information ☐ Very much information

**6. Have you decided the kind of dialysis you would want to receive if it becomes necessary?**

☐ Hemodialysis

☐ Peritoneal dialysis

☐ I will never want to start dialysis regardless of my renal function

☐ I don't know

**7. Whose opinion has been most important in making the decision about whether to have HD, PD or don't have dialysis at all?**

☐ That of my kidney doctor

☐ That of my nurse

☐ Mine

☐ That of my family or friends

**8. Have you been told by your doctor that it is time to start dialysis?**

- ☐ Yes, and I followed this advice (dialysis is started or planned at short notice)
- ☐ Yes, but I have decided to wait
- ☐ No
