# Supplementary material for: Converting from face-to-face to postal follow-up and its effects on participant retention, response rates and errors: lessons from the EQUAL study in the UK
Source: BMC Med Res Methodol. 2022 Feb 11;22:44. doi: 10.1186/s12874-021-01453-0 (PMC8832416; doi:10.1186/s12874-021-01453-0)
Supplement: Supplementary file 2 — Additional file 2: Infographic showing the content (black) and error rate (red) throughout the EFU patient questionnaire. [file 12874_2021_1453_MOESM2_ESM.pdf]

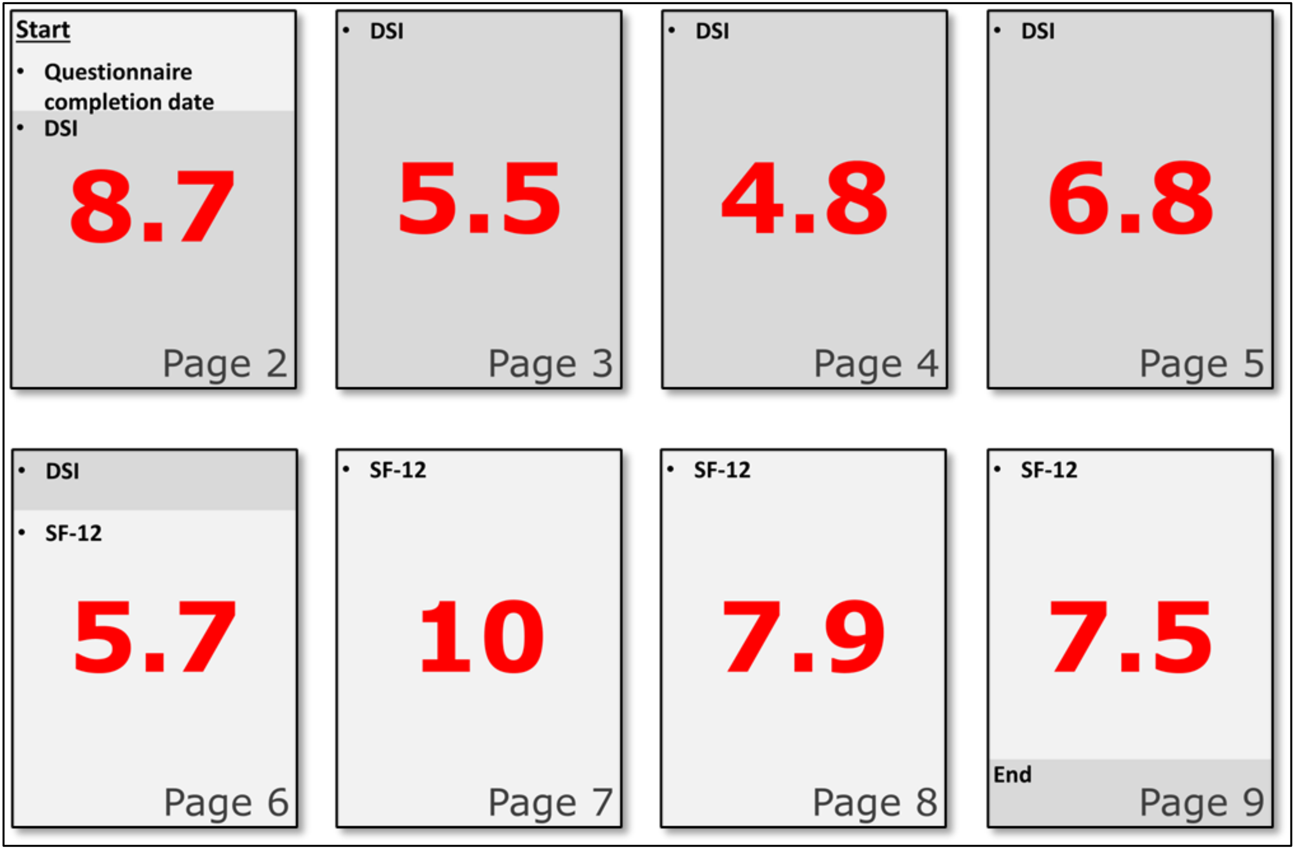
Start
Questionnaire completion date
DSI
8.7
Page 2
DSI
5.5
Page 3
DSI
4.8
Page 4
DSI
6.8
Page 5
DSI
SF-12
5.7
Page 6
SF-12
10
Page 7
SF-12
7.9
Page 8
SF-12
7.5
End
Page 9
